# Editorial: Onconephrology: evolving concepts and challenges

**DOI:** 10.3389/fneph.2025.1585605

**Published:** 2025-04-04

**Authors:** Rogerio Passos, Bruno Zawadzki, Etienne Macedo, Marcelino Durão, Fernanda Oliveira Coelho

**Affiliations:** ^1^ Medical Service Board, Davita Tratamento Renal, Rio de Janeiro, Brazil; ^2^ Intensive Care Medicine Department, Albert Einstein Israelite Hospital, São Paulo, São Paulo, Brazil; ^3^ Department of Medicine's Division of Nephrology-Hypertension, University of California, San Diego, La Jolla, CA, United States; ^4^ Nephrology Department, Albert Einstein Israelite Hospital, São Paulo, São Paulo, Brazil

**Keywords:** onconephrology, acute kidney injury, chronic kidney disease, monoclonal gammopathy of renal significance, nephrotoxicity, cancer treatment

The rapid evolution of cancer therapies—from conventional chemotherapy to targeted agents and immunotherapies—has significantly improved survival rates (1). However, these advances come with an increased risk of renal complications, which can limit treatment options and negatively affect patient outcomes. Acute kidney injury (AKI) and chronic kidney disease (CKD) are not merely secondary concerns; they can directly compromise cancer therapy by necessitating dose reductions, treatment delays, or even discontinuation. This underscores the essential role of the onco-nephrologist in optimizing cancer treatment while mitigating renal risks (2).

## The burden of kidney disorders in cancer patients

Renal complications are common among cancer patients, including AKI, CKD, electrolyte disturbances, acid-base imbalances, proteinuria, and hypertension. The incidence of AKI in this population is particularly high, often warranting nephrology consultation. The underlying malignancy and its treatment frequently contribute to renal dysfunction. For example, renal cell carcinoma (RCC) and multiple myeloma are associated with distinct mechanisms of kidney injury (3).

The evaluation of AKI in cancer patients follows the standard framework of prerenal, intrinsic, and postrenal causes. However, malignancy-associated factors, such as obstructive uropathy in bladder, prostate, and cervical cancers, are more prevalent. Additionally, hematologic malignancies like Burkitt lymphoma can induce AKI through tumor lysis syndrome or ureteral compression from retroperitoneal lymphadenopathy. Early nephrological assessment enables timely intervention, reducing the likelihood of treatment modifications due to kidney-related complications. Patients with cancer who develop AKI face prolonged hospitalizations, increased mortality, and worse oncologic outcomes, emphasizing the importance of early recognition and intervention (4).

## Nephrotoxicity in cancer treatment: established risks and emerging concerns

This review highlights key studies at the intersection of oncology and nephrology. As the landscape of cancer treatment continues to evolve, an increasing number of targeted therapies are being introduced, each with distinct renal toxicity profiles. While VEGF inhibitors have long been recognized for their association with endothelial dysfunction, thrombotic microangiopathy, and proteinuria, newer agents continue to reveal unexpected nephrotoxic effects (5). This highlights the importance of post-marketing surveillance in identifying and mitigating renal complications associated with emerging cancer therapies. One study examined the nephrotoxicity of poly (ADP-ribose) polymerase inhibitors (PARPis) using real-world pharmacovigilance data. Among nearly 1,700 reported renal adverse events, veliparib exhibited the strongest association with kidney injury, leading to hospitalization in nearly 30% of cases. These findings highlight the necessity of early detection and intervention to ensure patients can continue effective therapies without compromising renal function (Xu et al.).

Similarly, immune-based therapies, including immune checkpoint inhibitors (ICIs) such as PD-1/PD-L1 and CTLA-4 inhibitors, as well as chimeric antigen receptor (CAR) T-cell therapy, introduce new renal challenges. ICIs have emerged as a major cause of acute interstitial nephritis (AIN), often necessitating renal biopsy for definitive diagnosis. The loss of immune tolerance induced by ICIs leads to T-cell–mediated tubular injury, making them frequent causes of nephrology consultations in the oncohematologic setting. As with other immune-related adverse events, early recognition and timely corticosteroid therapy are essential to mitigating renal damage and avoiding long-term sequelae (6). Similarly, the profound immune activation triggered by chimeric antigen receptor (CAR) T-cell therapy, a groundbreaking advancement for refractory hematologic malignancies, also introduces significant renal challenges, including cytokine-mediated AKI. Effective renal management is critical for ensuring that patients can safely continue these transformative therapies, maximizing their oncologic benefits (Salvino et al.).

Nephrotoxicity is not limited to novel agents. Traditional chemotherapeutics, particularly platinum-based compounds, remain integral to cancer treatment but carry significant renal risks. Preventive strategies such as hydration protocols, nephroprotective measures, and close renal surveillance can reduce toxicity and sustain treatment efficacy (Lyrio et al.).

## Monoclonal gammopathy and kidney disease

Monoclonal gammopathy of renal significance (MGRS) represents a unique challenge, as monoclonal proteins can directly damage the kidneys, leading to progressive dysfunction. Unlike monoclonal gammopathy of undetermined significance (MGUS) or smoldering myeloma, where treatment is often deferred in the absence of malignancy, MGRS requires early clone-directed therapy to prevent irreversible renal damage. Without prompt recognition and intervention—often guided by an onco-nephrologist—patients are at an elevated risk of CKD and progression to end-stage renal disease (Shankar and Yadla).

## Precision medicine and renal oncology: the role of molecular insights

Advances in precision oncology increasingly highlight the need for integrating molecular diagnostics into clinical decision-making, allowing for individualized treatment strategies and improved patient outcomes. One study within this field explored RNA sequencing in RCC, identifying key prognostic biomarkers, such as MMP9, IFNG, and PGF, which correlate with survival outcomes. These markers may serve as valuable tools for refining therapeutic strategies (Verma et al.). Understanding these molecular mechanisms is crucial not only for oncologic prognosis but also for optimizing renal management in patients with RCC and other malignancies that affect kidney function.

## Challenges and future directions in onconephrology

The integration of nephrology expertise into oncology practice presents multiple challenges, from managing treatment-related nephrotoxicity to understanding the molecular basis of kidney involvement in cancer. Key barriers include limited awareness of onconephrology, a lack of specialized training programs, and insufficient collaboration between oncologists and nephrologists. Addressing these issues requires expanded education, interdisciplinary cooperation, and refinement of clinical guidelines to enhance renal care in cancer patients. The **Table 1** outlines key barriers and proposed solutions to enhance the integration of nephrology expertise into oncology, thereby improving both kidney-related and overall patient outcomes (4).

**Table 1:** Onconephrology challenges and proposed solutions.

Challenge	Proposed Solution
Fragmentation of Guidelines	Development of multidisciplinary consensus statements to standardize recommendations (2)
Exclusion of CKD patients from clinical trials	Advocacy for trial designs that include patients with kidney disease to improve evidence-based care (7)
Limitations of serum creatinine in GFR assessment	Implementation of Cystatin C-based GFR estimation while working toward laboratory standardization and specific biomarkers (8)
Lack of nephrology training among oncologists	Expansion of educational initiatives and collaborative programs, such as Onconephrotoxin Library Collaboration (OLIC) (9)
Variability in cancer treatments and renal toxicity	Utilization of real-world data and precision medicine approaches to tailor renal management strategies (10)

## The onco-nephrologist: a key partner in cancer care

To establish onconephrology as a vital and distinct specialty, continued investment in research, education, and collaboration is essential. Expanding specialized training programs, integrating nephrology expertise into oncology teams, and refining clinical guidelines will empower onco-nephrologists to address the complex renal challenges faced by cancer patients. Strengthening the foundation of onconephrology will not only improve patient outcomes but also ensure that kidney-related complications do not become a barrier to effective, life-saving cancer treatments (2, 3).

## References

[1] Falzone L Salomone S Libra M. Evolution of cancer pharmacological treatments at the turn of the third millennium. Front Pharmacol. (2018) 9:1300. doi: 10.3389/fphar.2018.01300 30483135 PMC6243123

[2] Porta C Bamias A Danesh FR Dębska-Ślizień A Gallieni M Gertz MA. KDIGO Controversies Conference on onco-nephrology: understanding kidney impairment and solid-organ Malignancies, and managing kidney cancer. Kidney Int. (2020) 98:1108–19. doi: 10.1016/j.kint.2020.06.046 33126977

[3] Brito GA Caires RA Coelho FO Campos MFT Cunha DFD Costalonga EC. Kidney care in patients with cancer: perspectives from the onconephrology committee of the Brazilian Society of Nephrology. Rev Assoc Med Bras (1992). (2024) 70:e2024S121. doi: 10.1590/1806-9282.2024S121 38865541 PMC11164260

[4] Hanna PE Chowdhury R Solhjou Z Gupta S Jhaveri KD. Challenges for optimal care in onconephrology. Nephrol Dial Transplant. (2024) 39:167–9. doi: 10.1093/ndt/gfad160 PMC10828197 37442629

[5] Kala J Salman LA Geara AS Izzedine H. Nephrotoxicity from molecularly targeted chemotherapeutic agents. Adv Chronic Kidney Dis. (2021) 28:415–428.e1. doi: 10.1053/j.ackd.2021.09.003 35190108

[6] Miao J Sise ME Herrmann SM. Immune checkpoint inhibitor related nephrotoxicity: Advances in clinicopathologic features, noninvasive approaches, and therapeutic strategy and rechallenge. Front Nephrol. (2022) 2:1017921. doi: 10.3389/fneph.2022.1017921 37674988 PMC10479679

[7] Inker LA Grams ME Guðmundsdóttir H McEwan P Friedman R Thompson A. Clinical trial considerations in developing treatments for early stages of common, chronic kidney diseases: A scientific workshop cosponsored by the national kidney foundation and the US food and drug administration. Am J Kidney Dis. (2022) 80:513–26. doi: 10.1053/j.ajkd.2022.03.011 35970679

[8] Inker LA Eneanya ND Coresh J Tighiouart H Wang D Sang Y. New creatinine- and cystatin C-based equations to estimate GFR without race. N Engl J Med. (2021) 385:1737–49. doi: 10.1056/NEJMoa2102953 PMC8822996 34554658

[9] Bonilla M Geara AS. Onconephrology fellowship training: current status and future outlook. Kidney360. (2024) 5:1380–5. doi: 10.34067/KID.0000000000000495 PMC11441803 39325593

[10] Sharma R Kannourakis G Prithviraj P Ahmed N. Precision medicine: an optimal approach to patient care in renal cell carcinoma. Front Med (Lausanne). (2022) 9:766869. doi: 10.3389/fmed.2022.766869 35775004 PMC9237320

